# Self-Assembly DBS Nanofibrils on Solution-Blown Nanofibers as Hierarchical Ion-Conducting Pathway for Direct Methanol Fuel Cells

**DOI:** 10.3390/polym10091037

**Published:** 2018-09-19

**Authors:** Hang Wang, Xiangxiang Li, Xiaojie Li, Xi Feng, Weimin Kang, Xianlin Xu, Xupin Zhuang, Bowen Cheng

**Affiliations:** 1 State Key Laboratory of Separation Membranes and Membrane Processes, Tianjin Polytechnic University, Tianjin 300387, China; whang_tjpu@yeah.net (H.W.); lixiaojie@tjpu.edu.cn (X.L.); 2 College of Textile, Tianjin Polytechnic University, Tianjin 300387, China; 15822369589@163.com (X.L.); kweimin@126.com (W.K.); xianlinxu@163.com (X.X.); 3 Department of Industrial Design, Yanshan University, Qinhuang Dao 066004, China; 15620697150@yeah.net

**Keywords:** hierarchical nanofiber, proton exchange membrane, SPES, DBS, solution blowing

## Abstract

In this work, we reported a novel proton exchange membrane (PEM) with an ion-conducting pathway. The hierarchical nanofiber structure was prepared via in situ self-assembling 1,3:2,4-dibenzylidene-d-sorbitol (DBS) supramolecular fibrils on solution-blown, sulfonated poly (ether sulfone) (SPES) nanofiber, after which the composite PEM was prepared by incorporating hierarchical nanofiber into the chitosan polymer matrix. Then, the effects of incorporating the hierarchical nanofiber structure on the thermal stability, water uptake, dimensional stability, proton conductivity, and methanol permeability of the composite membranes were investigated. The results show that incorporation of hierarchical nanofiber improves the water uptake, proton conductivity, and methanol permeability of the membranes. Furthermore, the composite membrane with 50% hierarchical nanofibers exhibited the highest proton conductivity of 0.115 S cm^−1^ (80 °C), which was 69.12% higher than the values of pure chitosan membrane. The self-assembly allows us to generate hierarchical nanofiber among the interfiber voids, and this structure can provide potential benefits for the preparation of high-performance PEMs.

## 1. Introduction

Nowadays, the direct methanol fuel cells (DMFCs) are considered as one of the promising green, reliable, and efficient power sources for portable and automotive applications due to their direct conversion of chemical energy to electricity [1]. Proton exchange membranes (PEMs), which selectively conduct protons and separate fuel, are the key components of DMFCs [2,3]. The current PEMs suffer some serious problems, including high cost, fuel penetration, and so forth, restricting their wide applications in DMFCs [4,5]. Therefore, alternatives to polymer membranes have been developed.

Among these methods, the composite PEMs based on nanofiber have attracted great attention due to the following reasons: (i) The nanofiber can act primarily as a mechanical support; (ii) the interactions between the functional nanofiber’s surface and matrix components can induce the formation of low-energy-barrier proton-hopping pathways [6]; (iii) and the nanofiber usually acts as a physical barrier layer to increase the tortuosity for fuel [7]. In general, the nanofiber composite PEMs were prepared via the combination of a nanofiber and ionomer matrix. Tanaka et al. [8] designed and synthesized phytic acid-doped polybenzimidazole nanofibers and filled the void space of nanofiber with Nafion. The resulting membrane was reported to present superior proton conductivity compared to the recast Nafion membrane at low relative humidity. The authors attributed the improved proton conductivity to the construction of the acid-condensed layer near the nanofibers via the acid–base interaction. Klose et al. [9] electrospun sulfonated poly(ether ketone) directly onto gas-diffusion electrodes and filled it with Nafion with direct membrane deposition, resulting in a 12 mm-thin membrane. The power density of the fuel cell with the prepared membrane was able to reach 2.04 W/cm^2^. In our previous work, we investigated the effects of different nanofiber, including sulfonated poly(ether ether ketone) [10], SiO_2_ [11], and poly(vinylidene fluoride) [12] on the performance of Nafion. All the results showed that the introduction of nanofiber could improve the performance of PEMs. Therefore, nanofiber has shown great promise in the preparation of materials for PEMs. Furthermore, the improvement of the orientation, distribution, and structure of the nanofiber in the membrane is accepted as being one of the most important strategies to promote interconnection between proton conductive channels and barrier tortuosity for methanol.

Inspired by leaf veins and blood vessels found in biological circulatory systems, hierarchical nanostructure materials have received extensive attention due to their unique structures, ability to enhance specific areas, and ample internal cavities [13,14,15]. The distinctive feature of this hierarchical nanofiber structure (HS) is their multi-level diameter, similar to leaf veins and blood vessels. In our previous work, we successfully explored the preparation of the tree-like nanofiber with trunk and branch fibers via electrospinning [13,16,17,18]. Recently, the self-assembly of low-molecular-weight gelators (LMWGs) in organic liquids have drawn great attention due to their tremendous potential as building blocks of bottom-up organic nano- and micro-scale devices [19]. Mativetsky et al. [20] prepared entangled networks of nanofibers based on donor–acceptor dyad architectures. In another work, Wakahara et al. [21] constructed supramolecular nanosheets comprising 1:1 fullerene/cobalt porphyrin by a simple liquid–liquid interfacial precipitation method. Therefore, the self-assembly can be an effective method to organize functional molecules into nanoarchitectures on nanofiber substrate to form a hierarchical nanofiber structureHS.

Chitosan (CS) consists of hydroxyl and amine functional groups, which is a promising candidate to act as polymer host for PEMs due to its excellent biocompatibility, high hydrophilicity, and low methanol permeability [22,23]. Furthermore, the acid–base pairs can promote proton migration via the Grotthuss mechanism [24,25]. Solution-blowing is an innovative nanofiber preparation technology which is recognized as a promising and scalable approach for the production of nanofibers [26,27,28]. In this process, the solution streams in the SBS process are stretched to ultra-thin jets by high-speed gas flow around the nozzle. Therefore, solution-blown nanofibers are commonly curled into three dimensions and closely entangled, showing three-dimensional curls. Moreover, the self-assembly nanoarchitectures inside the nanofiber void can provide multiple generations of nanochannels for proton transport and good shielding properties for the separation of methanol. These hierarchical structures mimicking leaf veins and blood vessels could be used as promising materials for PEMs. Therefore, in this work, we first present a novel preparation method of hierarchical nanofibrous mat via in situ self-assembling 1,3:2,4-dibenzylidene-d-sorbitol (DBS) supramolecular fibrils on solution-blown sulfonated poly (ether sulfone) (SPES) nanofiber. Then, the dense PEMs were prepared via filling pores of hierarchical nanofibers (acidic skeleton) with CS solution (base matrix). The effects of the hierarchical nanofiber on the as-prepared membranes were extensively evaluated in terms of dimensional stability, WU, methanol permeability, and proton conductivity.

## 2. Materials and Methods 

DBS (≥99.5%) was purchased from Georgia Think Technology Co., Ltd. (Tianjin, China). *N*,*N*-dimethylformamide (DMF) and N-butyl alcohol were purchased from Aladdin Co., Ltd. (Shanghai, China). PES was supplied by the Changchun Institute of Applied Chemistry (Changchun, Jilin, China).

The detailed synthetic method of SPES has been shown in our previous work [29]. The SPES nanofiber with hierarchical structures (DBS/SPES) was obtained according to the following methods: 35% SPES-DMF solution was chosen as the spinning solution, and then loaded into a syringe and squeezed out by a syringe pump at a speed of 8 mL h^−1^ through a needle with an inner diameter of 0.38 mm. The solution stream was blown and drafted by high-velocity airflow with 0.2 MPa air pressure. The SPES nanofiber mats were collected at a distance of 60 cm, and dried in a vacuum at 60 °C for 12 h to remove the residual solvent. DBS was dissolved in n-butyl alcohol to obtain a 0.28% solution. Each individual solution was filled into an immersion bath at a constant temperature of 80 °C. Then, the nanofiber mats were immersed vertically in the above DBS solution for 38 s. The soaked SPES nanofiber mat was removed and dried in a vacuum at 50 °C for 24 h to remove the immersed solvent. 

In order to prepare dense and compatible composite membranes, the voids of the DBS/SPES were further filled with chitosan. DBS/SPES was immersed in CS solution and dried at 50 °C for 8 h, followed by immersion in a 4% NaOH solution for 45 min. Then, the mat was washed with deionized water for neutralization. Finally, the membranes were dried at 80 °C for 12 h in a vacuum drying oven. The as-prepared composite membranes were designated as CS/HS-n (*n* = 40, 50, 60), where n represents the mass fraction of nanofibers. For comparison, a pure CS membrane was also prepared using the aforementioned procedure, and the thicknesses of all the membranes were approximately 120 μm. The characterization method is shown in the Supplementary Files.

## 3. Results and Discussions

**Figure 1:**
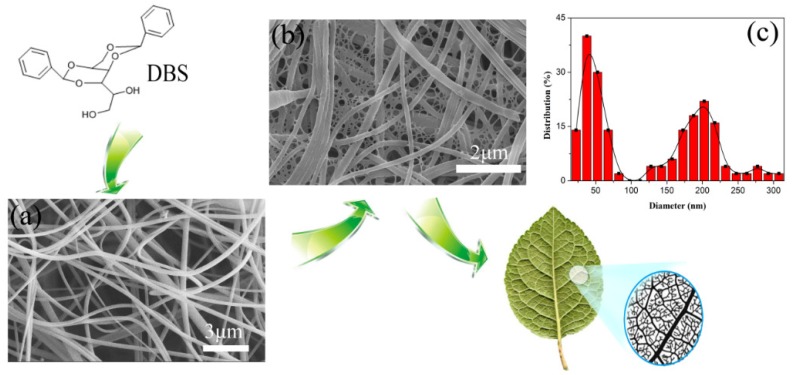
SEM of (**a**) sulfonated poly (ether sulfone) (SPES) solution-blown nanofibers and (**b**) 1,3:2,4-dibenzylidene-d-sorbitol (DBS)/SPES nanofibers; (**c**) the corresponding diameter distribution map of DBS/SPES.

Hydrogen bonding is the major driving force for the DBS self-assembly. Specifically, the primary alcohol group is intermolecularly hydrogen-bonded to an acetal oxygen-supporting self-assembly, and the phenyl groups are ordered side-by-side around the aggregate axis, and then the DBS nanofibers are formed. Figure 1a shows the Scanning electron microscopy (SEM) images of SPES solution-blown nanofibers, and DBS/SPES and their corresponding diameter distribution map. The SEM images revealed that the SPES solution-blown nanofiber had smooth surfaces and uniform fiber structures with a main diameter range of 150–250 nm. The morphology and structure of the DBS/SPES nanofiber were similar to leaf veins, as illustrated in Figure 1b. DBS nanofibers were successfully homogeneously generated among the interfiber voids. The DBS nanofibers had rather smaller diameters than SPES nanofibers, with a main diameter range of 30–70 nm. Moreover, the DBS/SPES nanofibers showed obvious hierarchical distribution characteristics, as shown in the diameter distribution map.

**Figure 2:**
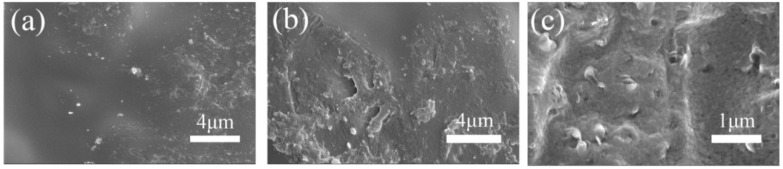
SEM images of (**a**) the chitosan (CS) membrane surface; and (**b**) SPES/HS-50% surface and (**c**) SPES/HS-50% cross-sections.

The surface and cross-section of the composite membrane is shown in Figure 2b,c. The images revealed that the composite membranes possessed a relatively dense and uniform structure. The nanofiber was finely embedded in the CS matrix, and no significant defects were observed between the nanofiber and the CS matrix phase. This phenomenon indicated that a compact composite membrane was prepared successfully.

The thermal stability of a PEM is a key property for its durability during fuel cell operations [22]. The decomposition profiles differed between the pure CS membrane and CS/HS membranes. Two main degradation stages were noticed in the CS membrane, whereas three stages of degradation were shown in the thermogravimetric analysis (TGA) analysis. The first instance of weight loss below 150 °C for all the samples was attributed to the evaporation of adsorbed water and residual solvent. The second step of the weight loss for the pure CS membrane was observed from 210 °C to 350 °C, which was the result of degradation of the carboxyl groups, amino groups, and CS main chains. However, the second degradation stage for the composite membrane started from approximately 280 °C due to the degradation of CS main chains and sulfonic acid groups in nanofiber. This result is probably due to the strong interaction between carboxyl groups and amino groups in CS and the sulfonic acid groups in the nanofiber, increasing the thermal stability of carboxyl groups and amino groups. Therefore, the presence of the DBS/SPES delays the degradation of CS membranes, leading to an improvement in the membrane’s thermal stability. The third weight-loss region for the composite membrane above 430 °C corresponds to the degradation of main polymer chain of SPES. Furthermore, the residual carbon value was consistent with the percentage of the nanofiber in membrane.

**Figure 3:**
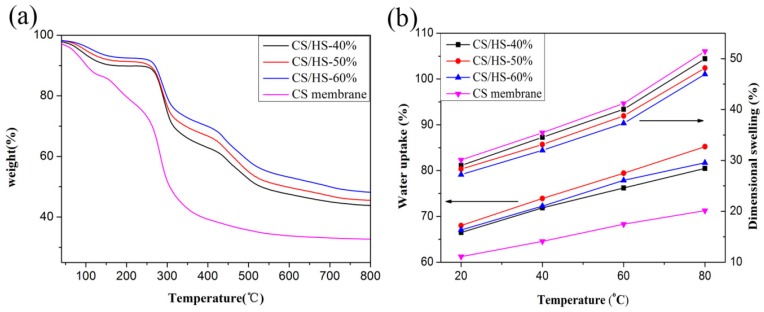
(**a**) TGA curves; (**b**) water uptake and dimensional swelling of all the samples.

The water uptake (WU) and dimensional swelling (DS) properties of the membranes at different temperatures are shown in Figure 3b. As shown in Figure 3b, WU and DS increased with the increasing temperature. The WU of all the composite membranes was higher than that of the pure CS membrane—however, their DS values were lower. Furthermore, the DS decreased with the increasing content of the DBS/SPES nanofiber. This result was expected, because (i) the hierarchical nanofiber skeleton kept the composite membrane structure together and prevented increases in size; and (ii) the incorporation of hierarchical nanofiber into the CS polymer matrix led to the formation of electrostatic forces between the functional groups on nanofiber and hydroxyl, ether, and amino groups in polymer [25,30]. CS/HS-50% showed the best WU in all the membranes, and this result indicates that the hierarchical nanofiber was a double-edged sword, because (i) it contained many polar groups along the backbone chain, which made it able to form hydrogen bonds with the functional groups of CS to increase the WU; (ii) and the excess content of nanofiber could not attract and retain more water inside the composite membrane due to the low matrix content. However, the increasing content of nanofiber can effectively keep the structure of composite membrane stable. Therefore, the DS decreased with the increasing content of nanofiber.

**Figure 4:**
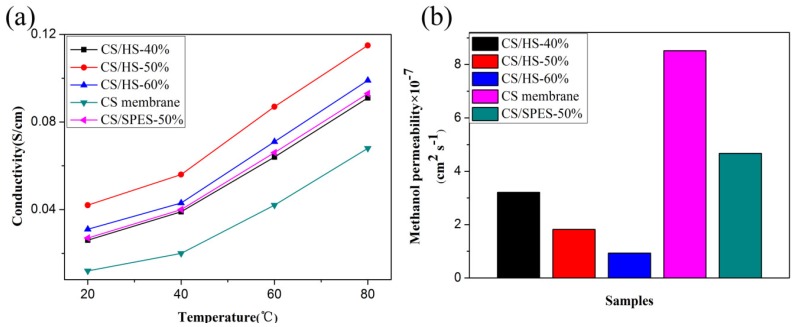
(**a**) Proton conductivity and (**b**) methanol permeability of pure CS and composite membranes.

The proton conductivity of the membranes measured at different temperatures and 100% RH is displayed in Figure 4a. Obviously, the pure CS membrane showed the lowest proton conductivity of 0.012 S cm^−1^ at 20 °C, and all the composite membranes showed higher proton conductivity than the pure CS membrane. The proton conductivity of the composite membrane showed a similar trend with the result of WU, and the CS/HS-50% displayed the highest proton conductivity of 0.115 S cm^−1^ at 80 °C. Furthermore, the proton conductivity of the composite membrane containing 50% pure SPES nanofiber (CS/SPES-50%) was also characterized for comparison. The proton conductivity of CS/SPES-50% was much lower than that of CS/HS-50%, not even that of CS/HS-60%. These results are likely due to the following aspects: (i) DBS/SPES components, including DBS and SPES, which contains massive functional groups (–OH and –SO_3_H) give rise to passable hydrophilic regions; (ii) the hierarchical network structure can form more effective proton channels for the transportation of protons than pure nanofiber; (iii) and the acidic –SO_3_H groups in SPES and basic –NH_2_ groups in CS form the acid–base pair, and then the protons transfer continuously via the Grotthuss mechanism by acid–base pairs. The values of methanol permeability of the pure CS membrane and composite membranes are shown in Figure 4b. The methanol permeability of pure CS, CS/HS-40%, CS/HS-50%, CS/HS-60%, and CS/SPES-50% was 8.52, 3.21, 1.82, 0.93, and 4.67 × 10^−7^ cm^2^ s^−1^, respectively. This result indicated that the methanol diffusivity across composite membranes was reduced significantly compared to the pure CS membrane. This result can be explained by the presence of the nanofiber structure, which can form barrier network structures to prevent methanol crossover. However, the CS/SPES-50% showed higher methanol permeability than the composite membrane containing hierarchical nanofiber. This result indicated that the self-assembly hierarchical nanofiber formed between SPES nanofibers had good shielding property for the separation of methanol to improve methanol permeability. In addition, it could be clearly observed that the proton conductivity and methanol permeability was improved simultaneously. A similar phenomenon was reported by Vijayalekshmi [31], and his membrane showed proton conductivity and methanol permeability of 4.8 × 10^−7^ cm^2^ s^−1^ and 2.85 × 10^−2^ S cm^−1^ at 30 °C, respectively. Compared with his membrane, the PEMs prepared in this study showed better performance due to the DBS/SPES nanofibers. Therefore, we can conclude that a high-performing PEM could be prepared by using the hierarchical nanofiber structure (HS).

## 4. Conclusions

In this study, we reported a novel preparation method of a hierarchical nanofiber structure, and then prepared a high-performance PEM by incorporating it into a CS polymer matrix. The performance of the composite membrane, including thermal stability, WU, DS, proton conductivity, and methanol permeability was improved due to the introduction of DBS/SPES. The CS/HS-50% displayed the highest proton conductivity of 0.115 S cm^−1^ at 80 °C higher than the CS membrane, for the acid–base interactions between SPES and CS may have helped to form the continuous ion-transporting channels. These results demonstrated that the hierarchical nanofiber structure has good potential for DMFC applications.

## Supplementary Materials

The following are available online at http://www.mdpi.com/2073-4360/10/9/1037/s1.
