# Supplementary material for: Self-Assembly DBS Nanofibrils on Solution-Blown Nanofibers as Hierarchical Ion-Conducting Pathway for Direct Methanol Fuel Cells
Source: Polymers (Basel). 2018 Sep 19;10(9):1037. doi: 10.3390/polym10091037 (PMC6403695; doi:10.3390/polym10091037)
Supplement: Supplementary file 1 [file polymers-10-01037-s001.pdf]

Supporting Information for:

Self-assembly DBS nanofibrils in solution-blown nanofibers as hierarchical ion-conducting pathway for direct methanol fuel cells

Hang Wang [1,2], Xiangxiang Li [2], Xiaojie Li [1], Xi Feng [3], Weimin Kang [2], Xianlin Xu [2], Xupin Zhuang [1,2,*], and Bowen Cheng [1,*]

[1] State Key Laboratory of Separation Membranes and Membrane Processes, Tianjin Polytechnic University, Tianjin300387, P.R.China; whang_tjpu@yeah.net (H.W.); lixiaojie@tjpu.edu.cn (X.L.)

[2] College of Textile, Tianjin Polytechnic University, Tianjin 300387, P.R.China; 15822369589@163.com (X.L); kweimin@126.com (W.K.); xianlinxu@163.com (X.X.)

[3] Department of Industrial Design, Yanshan University, Qinhuang dao, 066004, P.R.China; 15620697150@yeah.net (F.X.)

* Correspondence: zhxupin@tjpu.edu.cn (X. P. Zhuang), bowen15@tjpu.edu.cn (B.W.Cheng); Tel.: +86-22-83955353

Experimental Sections

Characterization

The morphologies of the nanofibers and membranes were characterized using a scanning electron microscope (SEM, Hitachi S-4800, Japan) after 2 min gold coating (E1045, Hitachi ion sputter, Japan). Fiber diameter was determined from SEM micrographs using an Image-Pro plus analyzer (Ipwin32, Soft Imaging System). The thermal stability of PES and SPES was investigated by high resolution thermogravimetric analysis (TGA), which was performed using TGA instruments (STA409PC).

Water uptake (WU) and dimensional swelling (DS) were calculated using the following equations:

$$WU\ (\%) = (W_w - W_d)/W_d \times 100\% \quad (1)$$

$$DS\ (\%) = (A_w - A_d)/A_d \times 100\% \quad (2)$$

where W_w and W_d were the water swollen membrane weight and dry membrane weight and A_w and A_d were the water swollen membrane area and dry membrane area, respectively. The proton

conductivity of samples was measured using four electrodes. The proton conductivity was measured by electrochemical impedance spectroscopy using an electrochemical workstation (CHI604E) from 0.1 Hz to 100 kHz. The proton conductivity was calculated from the formula:

$$\sigma = L\,A^{-1}\,R^{-1} \qquad (3)$$

where *L*, *A*, and *R* are the distance between the reference-sensing electrodes, the cross sectional area, and the resistance of the membrane, respectively. The methanol diffusion coefficients were calculated through the formula:

$$\mathrm{DK} = \frac{L \cdot V_B \cdot C_{B(t)}}{A \cdot C_{A(t-t_0)}} \qquad (4)$$

where C_A and C_B are the methanol concentrations of methanol side (10 M) and water side, respectively; *A*, *L*, and V_B are the effective area, thickness of the membrane, and volume of the permeated compartment, respectively; *DK* is the methanol permeability; t is the end time, and t_0 is the start time. Methanol concentration was monitored by gas chromatography (Agilent 7820) equipped with a thermal conductivity detector (TCD) and a DB-624 column.
